# Mosaic pregnancy after transfer of a “euploid” blastocyst screened by DNA microarray

**DOI:** 10.1186/1757-2215-6-70

**Published:** 2013-10-08

**Authors:** Ghassan Haddad, Wenyin He, Jimmy Gill, Craig Witz, Cassie Wang, Khalied Kaskar, Weihua Wang

**Affiliations:** 1 Houston Fertility Institute, New Houston Health, 2500 Fondren Rd., Suite 350, Houston, TX 77063, USA; 2 Key Laboratory of Major Obstetrics Diseases of Guangdong Province, Guangzhou Medical University, Guangdong, China

**Keywords:** Mosaicism, Blastocyst, Pregnancy, Human

## Abstract

**Background:**

High proportions of human embryos produced by in vitro fertilization are aneuploidy and mosaic. DNA microarray is one of the most practical screening methods to select euploid embryos for transfer. However, mosaic pregnancy is still possible due to embryonic mosacism. Here we report a successful pregnancy after transfer of a mosaic blastocyst with euploid inner cell mass.

**Methods:**

A woman with a previous trisomy 13 pregnancy pursued infertility treatment with preimplantation genetic screening by a trophectoderm biopsy and DNA microarray. NimbleGen oligonucleotide DNA microarray was applied to biopsied samples from 13 blastocysts. A euploid blastocyst was transferred to the patient and subsequent prenatal cytogenetic tests were performed by FISH and/or G banding.

**Results:**

Following DNA microarray, it was found that 5 blastocysts were euploid and 8 were aneuploidy. Transfer of one euploid blastocyst resulted in a clinical pregnancy. Prenatal cytogenetic tests of samples biopsied from chorionic villi sample showed both trisomy 21 (47 XX, +21) and euploid (46, XX) cells. Further prenatal cytogenetic test with a sample from amniotic fluid indicated that all cells were euploid (46, XX). The pregnancy was continued and a healthy girl was delivered after 41 weeks of gestation.

**Conclusions:**

This is the first report to indicate a mosaic pregnancy after transfer of a “euploid” blastocyst that was screened by DNA microarray, and the case further confirms that mosaicism is present in human blastocysts produced by in vitro fertilization.

## Introduction

Mosaicism refers to the presence of two or more cell lines in an individual or tissue sample [1]. When mosaicism is found in cultured fetal cells, there may be problems in interpreting whether the fetus is truly mosaic and in determining the clinical significance of this apparent mosaicism. Mosaicism can be detected from chorionic villi sample (CVS) or amniotic fluid by cytogenetic tests, such as fluorescence in-situ hybridization (FISH), GTG banding and DNA microarray [2-4]. Mosaicism can either be true mosaicism and false mosaicism. It has been found that true mosaicism is usually associated with a high risk of mosaicism present in the fetus [5].

Mosaicism is sometimes present in the placenta but absent in the fetus and this genetic inconsistency within the conceptus is known as confined placental mosaicism [5]. It has been reported when a live born infant or fetus has non-mosaic trisomy 13 or trisomy 18, there is placental mosaicism with normal cell line and/or a trisomic cell line [6]. In this case, the percentage of placental cells with a normal karyotype ranges from 12% to 100% [7-9]. This data suggests that a normal or abnormal placenta may be able to maintain the pregnancy of a trisomy fetus. It also suggests that a mosaic placenta can support a pregnancy of a normal fetus.

Confirmation and interpretation of mosaicism are among the most difficult challenges in genetic counseling for prenatal diagnosis. Currently, accurate prediction of clinical outcome based on information describing mosaicism may be impossible. Thus further studies with different tissue samples (CVS, amniocentesis or cordocentesis) at different periods of gestation may provide a more complex evaluation [2,3,6-9].

High proportions of human embryos produced by in vitro fertilization (IVF) are not only aneuploidy, but also mosaic [10-13]. Recently, a normal pregnancy was established after transfer of a day 5 blastocyst that was aneuploidy on a day 3 embryo biopsy screened by DNA microarray [14]. We also found that mosaicism is highly present in human blastocysts and a high proportion of the mosaic blastocysts had euploid inner cell mass (ICM), suggesting that transfer of these embryos may produce normal babies [13]. This kind of mosaicism, called diploid-aneuploid mosaicism [15], has been detected in trisomic and monosomic spontaneous abortion [16]. Previous study with blastocysts produced by IVF also indicated a high diploid-aneuploid mosaicism although the study did not compare trophectoderm (TE) and ICM [15]. When we compared ICM and TE samples from IVF blastocysts, we found that most diploid-aneuploid blastocysts actually had euploid ICM [13]. Therefore, if inconsistent mosaicism is present in a blastocyst between TE and ICM or inside TE cells, the information obtained by TE biopsy may not represent the actual chromosomal information in the embryo [13]. For example, if a blastocyst has a euploid ICM but an aneuploid TE or mosaic TE, the test information may mislead the decision for the final disposition of the embryo. If the biopsied cells are aneuploid, the embryos are considered abnormal and would not be considered for transfer. Thus, the patients may lose the chance to become pregnant if all of the viable embryos demonstrate aneuploidy.

Based on previous studies, TE biopsy and DNA microarray can significantly increase chance of embryo implantation [13,17-19], which suggests that DNA microarray of human blastocysts can screen most of the embryos. However, it is still difficult to correctly test the embryos because of mosaicism. Here, we report a case in which microarray of biopsied TE indicated a euploid embryo, but prenatal cytogenetic tests indicated a trisomy 21 placenta. Further testing demonstrated a normal euploid fetus.

## Case presentation

A 38 year old woman previously had two IVF cycles and a total of 5 embryo transfers (two fresh transfers and three frozen embryo transfers). The patient conceived following the last transfer, but ultrasonography demonstrated that the fetus developed abnormally. Cytogenetic testing showed trisomy 13. After termination of the pregnancy, the patient decided to have a third IVF cycle with 23-pair chromosome preimplantation genetic screening (PGS).

**Figure 1:**
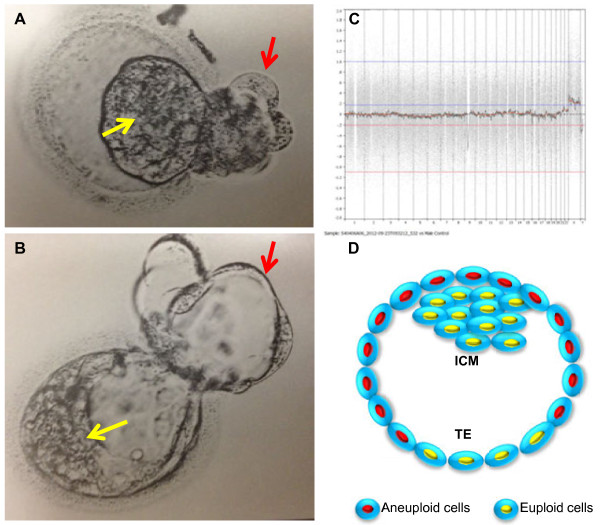
**Morphology and PGS chart of a blastocyst transferred. A)** Blastocyst after warming and **B)** same blastocyst after 2 hrs of culture. **C)** PGS chart of the sample biopsied from the blastocyst, showing 46, XX. **D)** Diagram of the blastocyst based on the PGS and prenatal cytogenetic tests, showing that the blastocyst has euploid inner cell mass, partial euploid trophectoderm (TE) and partial aneuploid (47, XX, +21) TE. Yellow arrows indicate the inner cell mass (ICM), and red arrows indicate the TE.

From the third IVF cycle, 25 eggs were retrieved and 22 were mature. After insemination and embryo culture, a total of 13 blastocysts were produced on Days 5 and 6. All blastocysts were biopsied for microarray with NimbleGen oligonucleotide DNA microarray platform [13]. The results of the PGS are shown in Table 1. Embryo number 7, a day 5 euploid hatching blastocyst (Figure 1) was transferred. Vaginal sonography demonstrated normal fetal growth in the first trimester.

**Table 1:** Microarray outcome of the biopsied trophectoderm samples

**Sample ID**	**DNA microarray ID**	**CMA results**	**Interpretation**	**Final disposition**
1C	091912-15-D5	46,XX,+16,-20	Aneuploidy	Still in storage
2C	091912-16-D5	45,XY,-22	Aneuploidy	Still in storage
3C	091912-17-D5	47,XY,+19	Aneuploidy	Still in storage
4C	091912-18-D5	47,XY,+22	Aneuploidy	Still in storage
5C	091912-19-D5	46,XY	Euploid	Still in storage
6C	091912-20-D5	46,XX	Euploid	Still in storage
7C	091912-21-D5	46,XX	Euploid	Transferred
8C	091912-22-D5	46,XY	Euploid	Still in storage
9C	091912-23-D5	46,XY	Euploid	Still in storage
10C	091912-24-D5	47,XY,+15	Aneuploid	Still in storage
11C	091912-25-D6	Multiple chromosomal abnormalities		Still in storage
12C	091912-26-D6	44,XY,-16,-21	Aneuploidy	Still in storage
13C	091912-27-D6	44,XY,-8,-9	Aneuploidy	Still in storage

As the patient was of advanced reproductive age, and because of a previous pregnancy with trisomy 13, the patient decided to have a prenatal cytogenetic testing. Prenatal cytogenetic test by 5 probes (13, 18, 21, X and Y) FISH of biopsied CVS samples showed that all cells (x100) had trisomy 21. However, GTG banding for chromosome analysis of cultured another CVS sample was euploid (46, XX). A microarrray was also performed on both CVS samples and it was showed a mixture of 46, XX and 47, XX, +21.

After the CVS results were made available, the DNA microarray from the trophectoderm biopsy was repeated and the result of a “46 XX” embryo was confirmed. Later at 15 weeks gestation, a sample of amniotic fluid was collected. FISH showed that all cells (x100) were 46, XX and GTG banding showed all cells (x30) were 46, XX too.

The pregnancy was continued with no sign of developmental abnormality and a healthy girl, weight 6 lbs 14 oz, was delivered by C section on June 6^th^, 2013.

## Discussion

Mosaic pregnancy has been previously observed in natural conception by prenatal diagnosis [5-9]. It has been found that some fetuses had normal chromosomes while the placenta had either normal and abnormal chromosomes, or completely abnormal chromosomes [5-8]. It is estimated that approximately 2% of viable pregnancies have this kind of mosaicism [9]. Furthermore, it is reported that there was about a 10% of risk of fetal mosaicism when placental mosaicism was diagnosed [9]. This indicates that most of the fetuses may be normal even if the placental mosaicism is found. Prevalence of chromosomal mosaicism in pregnancies from infertile couples was similar to those conceived naturally [20].

Previous studies by IVF and PGS indicated that mosaicism was mainly present in cleavage human embryos [10-12]. However, we recently found that high proportions of human blastocysts also are mosaic by DNA microarray of samples biopsied from ICM and two different locations of TE cells [13]. We found that some aneuploid/mosaic blastocysts screened by microarray had euploid ICM cells, thus transfer of these embryos would result in birth of healthy babies. The present case report, for the first time, provides the evidence that a healthy baby can be born if a blastocyst had a euploid ICM and mosaic TE.

Based on our previous findings [13], there are four different human embryonic mosaicisms: 1) embryos had aneuploid ICM and had both euploid and aneuploid TE cells; 2) embryos had aneuploid ICM and had euploid TE cells. These embryos are considered to be abnormal; 3) embryos had euploid ICM and aneuploidy TE cells and 4) embryos had euploid ICM and had both euploid and aneuploidy TE cells. Because these mosaic embryos have euploid ICM, transfer of these blastocysts should be able to establish a normal pregnancy. As shown in Figure 1, current pregnancy was established by transfer of an embryo that had euploid ICM and had both euploid and aneuploidy TE cells.

Although high frequencies of mosaicism have been reported in preimplantation human embryos, especially from women with advanced maternal age [10-12], the frequency of chromosomal mosaicism in spontaneous abortion specimens is still low [16]. This indicates that most of the mosaicism is placental mosaicism or placental aneuploidy and most mosaic embryos are lost if they have aneuploid ICM, resulting mainly from the first trimester spontaneous abortion [16]. It would appear that the rate of mosaic pregnancy is not related to infertility treatment and IVF [15]. It has been reported that there was no difference in the prevalence of mosaicism during the first trimester of pregnancies conceived naturally or with IVF [20]. However, it was found that diploid-aneuploid mosaicism rate is quite high in the blastocysts produced by IVF [15]. According to our previous study, four out of nine diploid-aneuploid mosaic blastocysts had euploid ICM [13]. These embryos are not used for transfer if PGS shows aneuploidy. Therefore, it was suggested that a second TE biopsy and microarray may be necessary if all embryos are aneuploid in a cohort of embryos from one IVF cycle and a normal embryo may be found for transfer. The second biopsy and microarray may be suitable only for embryos with a single chromosomal abnormality as it was found that some mosaic embryos with only a single chromosomal error had euploid ICM and no blastocyst with multiple chromosomal errors had euploid ICM [13]. Thus, we expect that blastocysts with multiple chromosomal errors may have significantly decreased chance of possessing a euploid ICM. Further studies remain necessary to confirm this hypothesis.

## Conclusions

In conclusion, this case report indicates that normal pregnancy and live birth of a healthy baby are possible if the diploid-aneuploid human mosaic blastocyst has a euploid ICM. A second TE biopsy may be necessary to find normal embryos from a cohort of mosaic blastocysts if all embryos are aneuploidy. Furthermore, the second TE biopsy and microarray likely should be limited to the embryos with a single chromosomal error.

### Consent

Written informed consent was obtained from the patient for IVF, PGS and publication of their data and any accompanying images after de-identification. When the patients signed the consents, they were aware that embryo biopsy and PGS are investigational procedures and the procedures were approved by the institutional research committee at Houston Fertility Institute. A copy of the written consent is available for the review by the Editor-in-Chief of this journal.

## Abbreviations

FISH: Fluorescence in-situ hybridization; CVS: Chorionic villi sample; ICM: Inner cell mass; PGS: Preimplantation genetic screening; TE: Trophectoderm; IVF: In vitro fertilization.

## Competing interests

The authors declare that they have no competing interests.

## Authors’ contributions

GH and JG performed patient consult, egg retrieval, embryo transfer and collect clinical data. WH, CW and WW wrote the manuscript. TW and KK performed embryo biopsy, embryo cryopreservation, embryo culture and transfer. All authors read and approved the final manuscript.

## References

[B1] Strachan T Read AP Human Molecular Genetics 1999 2 New York: Wiley–Liss

[B2] Alfirevic Z Mujezinovic F Sundberg K Amniocentesis and chorionic villus sampling for prenatal diagnosis Cochrane Database Syst Rev 2003 3 CD003252 12917956 10.1002/14651858.CD003252 PMC4171981

[B3] Miura S Miura K Masuzaki H Miyake N Yoshiura K Sosonkina N Microarray comparative genomic hybridization (CGH)-based prenatal diagnosis for chromosome abnormalities using cell-free fetal DNA in amniotic fluid J Human Genetics 2006 51 412 417 10.1007/s10038-006-0376-7 16622586

[B4] Wapner RJ Martin CL Levy B Ballif BC Eng CM Zachary JM Chromosomal microarray versus karyotyping for prenatal diagnosis N Engl J Med 2012 367 2175 2184 10.1056/NEJMoa1203382 23215555 PMC3549418

[B5] Kalousek DK Dill FJ Chromosomal mosaicism confined to the placenta in human conception Science 1983 221 665 667 10.1126/science.6867735 6867735

[B6] Goldberg JD Wohlferd MM Incidence and outcome of chromosomal mosaicism found at the time of chorionic villus sampling Am J Obstet Gynecol 1997 176 1349 1453 10.1016/S0002-9378(97)70356-9 9215195

[B7] Kalousek DK Vekemans M Confined placental mosaicism J Med Genet 1996 33 529 533 10.1136/jmg.33.7.529 8818935 PMC1050657

[B8] Ledbetter DH Zachary JM Simpson JL Golbus MS Pergament E Jackson L Cytogenetic results from the U.S. collaborative study on CVS Prenat Diagn 1992 12 317 345 10.1002/pd.1970120503 1523201

[B9] Phillips OP Tharapel AT Lerner JL Park VM Wachtel SS Shulman LP Risk of fetal mosaicism when placental mosaicism is diagnosed by chorionic villus sampling Am J Obstet Gynecol 1996 174 850 855 10.1016/S0002-9378(96)70312-5 8633655

[B10] Munne S Sandalinas M Escudero T Marquez C Cohen J Chromosome mosaicism in cleavage stage human embryos: evidence of a maternal age effect Reprod Biomed Online 2002 4 223 232 10.1016/S1472-6483(10)61810-X 12709271

[B11] Platteau P Staessen C Michiels A Van Steirteghem A Liebaers I Devroey P Preimplantation genetic diagnosis for anuploidy screening in women older than 37 years Fertil Steril 2005 84 319 324 10.1016/j.fertnstert.2005.02.019 16084871

[B12] Baart EB Martini E van den Berg I Macklon NS Galjaard RJ Fauser BC Van Opstal D Preimplantation genetic screening reveals a high incidence of aneuploidy and mosaicism in embryos from young women undergoing IVF Hum Reprod 2006 21 223 233 16155075 10.1093/humrep/dei291

[B13] Liu J Wang W Sun X Liu L Jin H Li M DNA microarray reveals that high proportions of human blastocysts from women of advanced maternal age are aneuploid and mosaic Biol Reprod 2012 87 1 9 10.1095/biolreprod.112.101691 23136294

[B14] Pabon JE Harton GL Seabaugh A Maitta R Srivastava RK Successful implantation and ongoing pregnancy of a single monosomy 16 preimplantation embryo: case Report Fertil Steril 2005 82 S333

[B15] Bielanska M Jin S Bernier M Tan SL Ao A Diploid-aneuploid mosaicism in human embryos cultured to the blastocyst stage Fertil Steril 2005 84 336 342 10.1016/j.fertnstert.2005.03.031 16084874

[B16] Hassold T Mosaic trisomies in human spontaneous abortion Hum Genet 1982 61 31 35 10.1007/BF00291327 7129422

[B17] Schoolcraft W Treff N Ferry K Stevens J Katz-Jaffe M Scott R First clinical application of SNP microarray based 24 chromosome aneuploidy screening of human blastocysts Fertil Steril 2010 94 S23 S24

[B18] Yang Z Liu J Collins J Salem SA Liu X Lyle SS Selection of single blastocysts for fresh transfer via standard morphology assessment alone and with array CGH for good prognosis IVF patients: results from a randomized pilot study Mol Cytogenet 2012 5 24 10.1186/1755-8166-5-24 22551456 PMC3403960

[B19] Liang L Wang CT Sun X Liu L Li M Witz C Identification of chromosomal errors in human preimplantation embryos with oligonucleotide DNA microarray PLoS One 2013 8 e61838 10.1371/journal.pone.0061838 23613950 PMC3628862

[B20] Huang A Adusumalli J Patel S Liem J Williams J Pisarska MD Prevalence of chromosomal mosaicism in pregnancies from couple with infertility Fertil Steril 2009 91 2355 2360 10.1016/j.fertnstert.2008.03.044 18554589

